# The Microbiome and Uremic Solutes

**DOI:** 10.3390/toxins14040245

**Published:** 2022-03-30

**Authors:** Nadim Zaidan, Lama Nazzal

**Affiliations:** 1 Faculty of Medicine, Saint Joseph University, Beyrouth 1004, Lebanon; nadim.zaidan@nyulangone.org; 2 Department of Medicine, Division of Nephrology, New York University Langone Health, New York, NY 10016, USA

**Keywords:** uremic solutes, gut microbiota, chronic kidney disease, cardiovascular risk

## Abstract

Uremic retention solutes, especially the protein-bound compounds, are toxic metabolites, difficult to eliminate with progressive renal functional decline. They are of particular interest because these uremic solutes are responsible for the pathogenesis of cardiovascular and chronic kidney diseases. Evidence suggests that the relation between uremic toxins, the microbiome, and its host is altered in patients with chronic kidney disease, with the colon’s motility, epithelial integrity, and absorptive properties also playing an important role. Studies found an alteration of the microbiota composition with differences in species proportion, diversity, and function. Since uremic toxins precursors are generated by the microbiota, multiple therapeutic options are currently being explored to address dysbiosis. While an oral adsorbent can decrease the transport of bacterial metabolites from the intestinal lumen to the blood, dietary measures, supplements (prebiotics, probiotics, and synbiotics), and antibiotics aim to target directly the gut microbiota composition. Innovative approaches, such as the modulation of bacterial enzymes, open new perspectives to decrease the plasma level of uremic toxins.

## 1. Introduction

Uremic retention solutes (URS), also known as uremic toxins, are defined as compounds that accumulate with declining renal function. Their classification depends on their chemical and physical properties. The middle molecules are distinguished from the small compounds by their higher molecular weight. Uremic retention solutes are further subdivided into small water-soluble solutes (such as urea) and protein-bound solutes (such as indoxyl sulfate and p-cresyl sulfate) [1].

These molecules are the mediators of the uremic syndrome, impacting multiple systems, especially the cardiovascular system, which accounts for the majority of mortality in end-stage kidney disease (ESKD) [2]. Since cardiovascular mortality in ESKD cannot be fully accounted for by traditional risk factors, there has been interest in identifying non-traditional factors. Uremic toxins represent one of the pathophysiological pathways linking uremia to cardiovascular disease, with observational studies suggesting a relationship between the plasma concentrations of protein-bound uremic toxins and the risk of cardiovascular disease (CVD) in patients with chronic kidney disease (CKD) [3].

## 2. Microbiome-Derived URS

**Figure 1:**
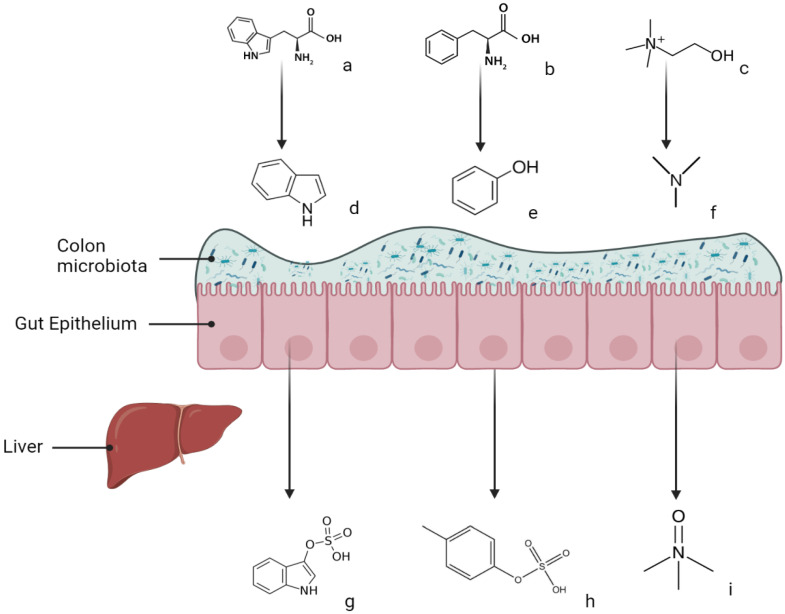
Simplified pathogenesis of three microbiome-derived URS. In the gut lumen, gut bacteria metabolize amino acids such as tryptophan (**a**) and phenylalanine (**b**) and dietary compounds such as choline (**c**). Tryptophan is metabolized into indole (**d**), phenylalanine into phenol (**e**), and choline into TMA (**f**). These URS precursors are then absorbed from the gut lumen into the bloodstream; once they reach the liver, they are conjugated with a sulfate (such as IS (**g**) and PCS (**h**)) or oxidized like TMA into TMAO (**i**) (created with BioRender.com, https://app.biorender.com/user/signin, Created 28 February 2022).

The most widely studied molecules in this class are indoxyl sulfate (IS) and p-cresyl sulfate (PCS) (molecular weights 213 and 188, respectively), which are inadequately cleared by conventional hemodialysis as they are protein-bound [4]. Both IS and PCS result from the processing of amino acids by the gut microbiota. Their pathogenesis is summarized in Figure 1.

Tryptophan, an IS precursor, can be metabolized by bacterial tryptophanase found in the genera Citrobacter, Proteus, or Escherichia, leading to indole [5], which is absorbed into the hepatic portal system to be oxidized by hepatic cytochrome p450-2E1 into indoxyl; it is then sulfated by a sulfotransferase to form IS [6]. IS is eliminated by the organic anion transporters OAT1 and OAT3 found in the renal tubules. IS is independently associated with cardiovascular (CV) events [7] and predicts CVD in advanced CKD [8]. In another multistep pathway, bacterial metabolism of tryptophan leads to indole-3-acetaldehyde, and its hepatic oxidation results in indole-3-acetic acid (IAA) [9]; this has essentially been described in *Bacteroides* spp. [10].

Anaerobic commensals such as Bacteroides, Lactobacillus, Clostridium, and Bifidobacterium generate phenols such as p-cresol from the amino acids tyrosine and phenylalanine [5]. P-cresol is then conjugated in the liver with either sulfate or glucuronide, leading, respectively, to PCS or p-cresyl glucuronide (PCG); both of these protein-bound compounds are eliminated through urinary secretion by the tubules [11]. In a meta-analysis (11 studies, 1572 patients with CKD or ESKD) [12], elevated free PCS was associated with a 16% and 28% increase in risks of all-cause mortality and CV events, respectively. Tyrosine is also a precursor to phenol via bacterial tyrosine phenol-lyase (TPL) [13]; phenol is then metabolized by the liver to phenyl sulfate (PS). PS correlated with albuminuria and its increase over time in a cohort of diabetic patients. PS was also shown to be toxic to podocytes in two murine models of diabetes [14].

Other gut-derived URS have also been implicated in CVD. Notably, trimethylamine N-oxide (TMAO) is a product of the metabolism of dietary D,L-carnitine and choline by commensal bacteria to trimethylamine (TMA) [15]. For instance, choline is metabolized via a bacterial glycyl radical enzyme (choline TMA-lyase) [16]. TMA is then absorbed and oxidized in the liver by the enzyme flavin monooxygenase-3 (FMO) to TMAO, which is a known toxin in both CKD and non-CKD patients [17]. An elevated serum concentration of TMAO was linked to an increased risk of adverse cardiovascular events [18]. Following 521 CKD patients for 5 years, Tang et al. found approximately a threefold increase in mortality when comparing the highest and lowest quartile of TMAO serum levels [19]. The thrombogenic effect of TMAO seems to be mediated by an upregulation of tissue factor as shown in animal studies [20].

In a study of 394 incident hemodialysis (HD) patients [21], free levels of four URS (IS, PCS, hippurate, and phenylacetylglutamine) were associated with a higher risk of CV morbidity and all-cause mortality. While this study also sought to demonstrate the synergistic toxicity of multiple URS by analyzing the outcomes using a “combined solutes index”, with those in the highest quintile associated with a 96% highest risk of death, combining only PCS and phenylacetylglutamine provided statistically similar results.

Besides the extensively studied phenols and indoles toxins, metabolomic profiling has led to the discovery of new URS, with Tanaka et al. identifying 120 solutes, 48 of which were novel compounds in their study comparing six HD patients with healthy subjects [22]. The largest proportion of these compounds consisted of modified amino acids and products of their metabolism. In a study by Mishima et al., the contribution of the colonic microbiome to URS accumulation was studied in CKD mice raised in a germ-free environment [23]. Glutarate, cholate, dimethylglycine, phenaceturate, gamma-guanidinobutyrate, and 2-hydroxypentanoate were among the 11 microbiome-derived solutes identified, which were further divided as originating from the gut microbiota either completely or partially (with the host or diets).

## 3. Uremic Toxins in Relation with the Gut Microbiome

### 3.1. The Colon in CKD Patients

Besides chemical classification, uremic toxins can be classified according to their origin. An addition, among multiple sources, the intestinal tract and specifically the colon plays a major role in uremic toxin production. Aronov et al. studied ESKD patients with (*n* = 9) and without a colon (*n* = 6), undergoing hemodialysis, and compared them to healthy individuals [24]. Using the differences in the magnitude of mass spectrometric features in these different populations, they showed the microbial origin of multiple uremic toxins, with 35 compounds absent or at a lower concentration in dialysis patients with colectomy. A more recent study by Mair et al. was able to specifically identify colon-derived uremic toxins by metabolomic analysis of plasma samples provided from patients with colectomies [25]. Comparison of urine and plasma samples from healthy controls (*n* = 17), patients with total colectomies (*n* = 12), and patients on HD (*n* = 14) identified 91 colon-derived solutes based on a ≥4-fold higher urine excretion rate in colectomy patients, 42 of which are conjugates (either by sulfation or glucuronidation). Sixty accumulated in HD patients to a degree greater than urea and were classified as URS; 12 of these 60 were characterized as modified amino acids and 7 of these 12 as phenylacetic acid (PAA) conjugates. PAA was previously identified by Tanaka et al. as a potential bacterial degradation product of phenylalanine and a colon-derived URS [22]. The analyzed solutes were mainly secreted by the tubules, a mechanism that physiologically offsets the limited glomerular filtration imposed by protein-binding [26].

The colon may be compared to a bioreactor, an interface between the resident bacteria constituting the gut microbiota and their environment and the host, with its histological and physiological characteristics playing an important role in uremic toxin accumulation.

CKD seems to alter this relationship, affecting the metabolism of compounds normally excreted by the kidney. Wikoff et al. [27] showed, by comparing germ-free and conventional rodents, an impact of the microbiota on multiple steps of uremic toxins pathogenesis. The plasma level of tryptophan and its metabolites were significantly lower in conventional mice, which can be attributed to the direct action of the gut microbiota. Even liver metabolism was influenced by the microbiota, with phenyl and p-cresol sulfate found only in conventional rodents and sulfation significantly lower for many compounds in germ-free animals. A recent study by Wang et al. shed light on how uremic toxins could, in turn, participate in the progression of renal failure [28]. When germ-free rodents with adenine induced CKD were transplanted with gut microbiota from healthy or ESKD donors, both plasma levels of URS and indicators of renal injury such as severe fibrosis and elevated creatinine and blood urea nitrogen (BUN) levels were observed. Interstitial fibrosis was also observed in animals exposed to a diet that led to a chronic elevation in TMAO [19].

### 3.2. The Gut Microbiota

Two species of the Clostridia class (*Eubacterium* spp. and *Faecalibacterium prausnitzii*, both Gram-positive anaerobes) and the Bacteroides genus from the Bacteroidia class (which is an obligate anaerobic Gram-negative bacteria) represent together at least two-thirds of the gut microbiota in healthy individuals [29,30]. The gut microbiome refers to the combined genetic potential of the gut microbiota, resulting in a mutualistic relation between bacteria and host [31]; an example of this relationship can be found in the metagenomic analysis of this ecological community. Analysis of bacterial DNA in the stools of the two healthy patients in the study of Gill et al. demonstrated that the gut microbiome has a remarkably augmented metabolism of numerous compounds such as amino acids and glycans while playing a major role in methanogenesis or detoxification of xenobiotics [32].

The gut microbiota diversity is altered in CKD; a potential explanation lies in urea secretion in the gastrointestinal tract, which leads to the formation of ammonia, with the increase in intestinal pH favoring the persistence of a state of dysbiosis [33]. An analysis of the fecal microbiota by Vaziri et al. showed an increase of the aerobic microbiota in hemodialysis patients, with Enterobacteria and Enterococci species increased a hundredfold [34]. Conducting analysis using phylogenetic microarrays, they also revealed an increase in the phyla Proteobacteria, Actinobacteria, and Firmicutes. In another study analyzing the microbiota of the lower intestine in patients with CKD, some families were found in lower numbers, such as the Lactobacillaceae and Prevotellaceae [35].

Identification of communities involved in URS production was actively pursued, leading to a better characterization of the taxa responsible for tyrosine, choline, and carnitine luminal metabolism. Two studies were first conducted in non-CKD populations. Aiming to identify bacteria that produce TMA, Rath et al. performed a metagenomic evaluation of the Human Microbiome Project (HMP) data [36]. In the screened samples, bacteria encoding for enzymes metabolizing choline (choline TMA lyase) and carnitine (carnitine oxygenase) were present in 71 and 27% of all the samples, respectively. Saito et al. cultured 153 taxa in tyrosine-enriched media, detecting p-cresol and phenol production in 36 and 23% of strains, respectively [37].

Stratifying patients according to the Kidney Disease Improving Global Outcome (KDIGO) CKD classification, Gryp et al. cultured fecal samples to identify taxa involved in URS pathogenesis [38]. After performing a taxonomic analysis, a metabolic test assessing bacterial ability to generate indolic and phenolic derivates from aromatic amino acids was used to define protein-bound uremic toxins (PBUT) generation ability. Of 148 isolated species, 92 were characterized as PBUT precursor-generating bacteria. The intestinal microbiota was altered in this CKD population with a decrease of anaerobic species. Kidney dysfunction was inversely correlated with the abundance of *Bifidobacterium* spp., *Roseburia* spp., and *F. prausnitzii*. Interestingly, the differences in specific species do not translate into differences on an ecological level. Kim et al. studied the microbiome’s diversity metrics in healthy (*n* = 46) and CKD patients (*n* = 103) [39]. CKD patients were divided into three categories: mild CKD (stages 1 and 2), moderate/severe CKD (stages 3 to 5), and HD patients. Although the URS measured positively correlated with the degree of kidney disease, neither alpha diversity using the Shannon index nor beta diversity using the Bray–Curtis dissimilarity index showed a difference between the four groups. Using the same diversity metrics in the evaluation of patients with early CKD, Sato et al. also did not find a significant difference [40].

The state of dysbiosis is manifested by increased production of gut metabolites by some of these ESKD-associated microbial species, as demonstrated by Wang et al. [28], with the effect size of the gut microbiome of ESKD patients accounting for, respectively, 31.3% and 39% of the serum and fecal metabolome variance. Beyond modifications at the metabolomic level, this study’s genetic analysis of key enzymes in uremic toxins pathogenesis showed that these genes were found more abundantly in patients with ESKD. Changes at the metagenomic levels in CKD patients were also described by Xu et al. [41]; notably, they used the metagenomic functional prediction software PICRUST to show gene variations for enzymes involved in bacterial metabolism of nutrients such as L-carnitine and choline.

### 3.3. Host–Microbiota Interaction

The integrity of the epithelial barrier plays a role in the regulation of host–bacteria homeostasis. Gut permeability is increased in the uremic state [33], with the disrupted tight junction leading to translocation of both bacteria and endotoxin in patients with CKD [42].

Gut motility is another important consideration in understanding the interplay between uremic toxins, the host, and the microbiota. In the ESKD population, constipation is extremely common; a meta-analysis by Zuvela et al. found that prevalence was more marked in peritoneal dialysis (14.2% to 90.3%) than hemodialysis (1.6% to 71.3%) [43]. Constipation was linked to both CVD mortality and CKD progression [44]. In a study by Sumida et al. following 3.5 million individuals with estimated glomerular filtration rate (eGFR) > 60 mL/min per 1.73 m^2^ over 7 years, constipated patients had a higher incidence of CKD and ESKD [45].

Constipation is multifactorial, with factors such as comorbidities, the medications used, metabolic abnormalities, and even uremic toxins and dysbiosis contributing to a pathological transit time in the gastrointestinal tract [46]. End products of the fermentation of plant-derived carbohydrates such as short-chain fatty acids (SCFA) have a stimulatory effect on the contractility of the ileum and colon; a reduction of these SCFA secondary to a decrease in the anaerobic flora could provide an explanation for constipation [47]. In a study by Parthasarathy et al. [48], the bacterial community of the colonic mucosa (but not the microbiota composing the stools) was associated with constipation: when comparing them with healthy controls, constipated patients exhibited a greater quantity of the Bacteroidetes phylum in the colonic mucosal microbiota. In animal studies, some pharmacological treatments of constipation had a direct impact on the gut microbiota. In an adenine-induced CKD mouse model treatment with a chloride channel activator (Lubiprostone) had a protective effect against renal damage while having a positive effect on bacterial communities of the rodent’s intestine with a recovery of Prevotellaceae and Lactobacillaceae family [49]. The use of Linaclotide, a guanylate cyclase C agonist, at high doses led to a decrease in TMAO and fibrosis in the kidneys and the heart [50].

## 4. Advances and Therapies Targeting the Microbiome in CKD to Control URS Levels

Delaying the progression of kidney failure plays an active role in the reduction in URS concentration. Eloot et al. suggest that pre-dialysis levels of URS do not depend on dialysis adequacy but rather on residual renal function [51]. A similar correlation was found in a study by Marquez et al. [26], where solute concentrations of 25 hemodialysis patients were measured to assess the contribution of residual function, which contributed more to the removal of protein-bound solutes than to the removal of urea. Elevated plasma levels were primarily found to be due to impaired kidney clearance and could not be attributed to differences in microbiota URS production since the levels of these URS and their precursors were unchanged in urine and stools [52].

Extracorporeal clearance of URS remains limited, especially in advanced CKD. Therefore, strategies that decrease absorption and reduce production and subsequent accumulation of URS for patients on dialysis become necessary.

### 4.1. Oral Adsorbent

AST-120 (Kremezin) is an orally administered adsorbent consisting of porous carbon microspheres. These particles adsorb precursors of the URS in the gut lumen, which allows them to be eliminated with the stools [53]. A systematic review and meta-analysis by Chen et al. assessed nine randomized controlled trials (RCT) involving a heterogeneous CKD population (from KDIGO stages 3 to 5) [54]. They found that despite its capacity to significantly decrease IS levels, its effect on clinical outcomes such as progressive renal failure and mortality are not clear-cut. For instance, while the Evaluating Prevention of Progression in CKD (EPPIC) trials result did not support the hypothesis that AST-120 could delay the progression of kidney failure, a post hoc study evaluating patients from the Unites States argues that subsequent studies in this specific population might show a clearer benefit [55,56]. Regarding the cardiovascular system, AST-120 was found to delay atherosclerosis in a uremic atherosclerosis mouse model by preventing the reduction of soluble fms-like tyrosine kinase 1 (Flt1) (an antiangiogenic factor whose expression is usually suppressed by uremic toxins in vitro) [57]. However, an RCT comparing AST-120 with magnesium oxide (MgOx) was prematurely stopped after the interim results clearly showed the benefit of MgOx on coronary calcification while AST-120 failed to significantly affect this endpoint [58]. An alternative to this drug, Renamezin, was first shown to decrease IS in a study involving 118 CKD patients followed for 8 weeks [59]. In a prospective cohort enrolling 1149 patients, Renamezin was assessed for compliance (76.8% of patients), safety (approximately 10% of patients reported gastro-intestinal adverse events), and efficacy (constant eGFR after 24 despite a significant creatinine increase of 0.2 mg/dL) [60].

### 4.2. Nutritional Measures

The type of diet is extremely important to influence the microbiome and try to decrease the serum URS levels. In a study by Patel et al. comparing vegetarians (*n* = 15) with omnivores (*n* = 11), the average excretion of PCS and IS was decreased in individuals with a restricted diet (respectively, 62% and 58% lower) [61]. The analysis of the respective diets showed a lower protein intake and a higher fiber intake in vegetarians. In a randomized controlled trial (RCT) comparing resistant (*n* = 28) and control starch (*n* = 28) supplementation, plasma IS and PCS were measured at the beginning of the study and after 6 weeks [62]. Patients in the resistant starch arm exhibited a significant decrease in the unbound-IS serum concentration and a decreasing trend in unbound-PCS. A diet rich in fiber in patients with CKD, while increasing the risk of hyperkalemia, should also be considered for constipation given its association with a decreased mortality risk. In a meta-analysis by Kelly et al., a healthy diet consisted of a higher fiber and lower red meat amounts [63]. Analysis of six of the seven studies established an association between a healthy diet and reduced all-cause mortality (adjusted relative risk of 0.73 with a 95% confidence interval between 0.63 and 0.83) but no impact on the risk of ESKD.

The Medika study aimed to evaluate the impact of a Mediterranean diet and protein restriction in a crossover study following 60 CKD patients for 18 months [64]. The investigators found that both diets were associated with lower levels of free and total IS and PCS than when patients were on a free diet. The follow-up to this study found that supplementing the Mediterranean diet with ketoanalogs further reduced these URS serum levels, although not as much as the Very Low Protein Diet [65].

Other nutritional measures focusing on supplementing patients with specific compounds are currently being explored. Resveratrol, a polyphenol commonly found in different types of berries, had a TMAO-reducing effect in mice fed choline, either one time or chronically over 1 month [66]. Mice receiving Resveratrol in their diet also had enrichment in Lactobacilli and Bifidobacteria; the ability of these mice’s cecal flora to convert choline to TMA in vitro was also significantly reduced. These promising findings could not be translated to the bedside, with trans-resveratrol unable to affect plasma level of PCS IS and IAA in a crossover trial where 20 CKD patients were allocated to either this supplement or placebo [67]. A curcumin-based additive was tested in 24 CKD patients [68]. After the patients were followed for half a year, no significant differences were found in the two URS (IS and PCS) and the majority of cytokines studied.

### 4.3. Supplements

Gut microbiota can also be modulated by the use of prebiotics or probiotics, used alone or in combination. During the last decade, three consensus statements were published on these supplements by the International Scientific Association for Probiotics and Prebiotics (ISAPP) to better define them and determine their scope. A fourth statement was issued concerning postbiotics. These supplements will be summarized in Table 1.

#### 4.3.1. Prebiotics

As defined by the 2017 ISAPP published statement, a prebiotic is a “substrate that is selectively utilized by host microorganisms conferring a health benefit” [69]. A review by Robertfroid et al. states that the majority of the data gathered on the effect of prebiotics in experimental and clinical studies was obtained using two categories of substrate: either the inulin-type fructans (ITF) or the galacto-oligosaccharides (GOS) [70]. This review notes that the first prebiotics available commercially were able to promote the growth of Bifidobacterium and, to a lesser extent Lactobacillus, while some studies witnessed a decrease in bacteria of the Clostridia genus.

The use of oligofructose-enriched inulin (p-inulin) has led to conflicting data in two studies involving hemodialysis patients [71,72]. In a study by Meijers et al. [71], the use of p-inulin during 4 weeks in 22 hemodialysis patients was associated with a decrease in PCS generation rates and serum concentrations but did not have an impact on IS generation or level. Raj et al. described a modification of the gut microbiome composition of 11 hemodialysis patients supplemented with p-inulin [72]. A change in the microbiome composition from pretreatment to post-treatment was evident (*p* = 0.004), but an effect of p-inulin on the level of IS, PCS, or TMAO was not evident.

Other types of prebiotics have been evaluated. An RCT with cross-over by Poesen et al. evaluating the effect of arabinoxylan oligosaccharides vs. placebo on colon-derived uremic toxins did not find significant differences in serum levels and renal excretion of IS, PCS, or TMAO in patients with CKD [73]. A pilot study investigating the impact of resistant starch in patients with ESKD receiving hemodialysis found that biomarkers of inflammation were reduced after 4 weeks of supplementation [74]. Unripe banana flour was studied in a crossover study of 43 ESKD patients treated with peritoneal dialysis. This prebiotic did not affect the URS (IS, PCS, and IAA) measured in the serum [75]. An RCT by Ramos et al. included nondiabetic patients with eGFR less than 45 L/min/1.73 m^2^ [76]. A total of 26 patients received a placebo and 24 patients were treated with fructo-oligosaccharide and the levels of URS were measured. There were no significant changes in IS and IAA, although a trend towards PCS reduction was described. A more recent CKD study followed 46 patients treated either with fructo-oligosaccharide or a placebo [77]. It demonstrated IL6 reduction and a same trend towards PCS reduction with 3 months of the prebiotic. Arterial rigidity evaluated using pulse wave velocity was not affected by the intervention.

#### 4.3.2. Probiotics

As defined by the most recent consensus conference of ISAPP on probiotics in 2013, the definition of a probiotic is that of “live microorganisms which when administered in adequate amounts confer a health benefit on the host” [78], with Bifidobacterium, Streptococci, and Lactobacilli among commonly used species. In animal studies [79,80], probiotic use was associated with a positive impact on the integrity of the gut epithelium. Hemodialysis patients receiving Bifidobacterium longum orally in gastroresistant capsules were found to have decreased homocysteine and IS plasma levels on pre-dialysis samples [81]. In a study by Ranganathan et al., the administration of probiotics (S. thermophilus, L. acidophilus, and B. longum) to 246 patients with CKD led to a decrease in BUN (and a non-statistically significant decreasing trend in creatinine and uric acid) [82]. In a placebo-controlled RCT, Liu et al. studied the effect of a probiotic supplement (a mixture of B. longum, L. acidophilus, and E. faecalis) on the microbiome. There were no significant changes in diversity, but there was a reduction in the level of several URS in the blood [83]. Lim et al. studied the effect of a mixture of two Lactobacilli with Lactococcus in 56 HD patients on metabolic and inflammatory parameters as well as plasma levels of PCS and IS [84]. After 24 weeks, PCS, metabolic, and inflammatory levels were unchanged, but IS significantly decreased in patients receiving the probiotic.

#### 4.3.3. Synbiotics

Following the latest ISAPP conference on synbiotics [85], their definition was updated to “a mixture comprising live microorganisms and substrate(s) selectively utilized by host microorganisms that confers a health benefit on the host”. Two RCTs conducted in 2014 evaluated synbiotic treatment in different populations of patients. In their pilot study, Guida et al. compared Probinul-neutro (a synbiotic consisting of a mixture of different Lactobacilli and Bifidobacteria with inulin and resistant starch) to placebo over 4 weeks in 30 patients with eGFR between 15 and 59 mL/min/1.73 m^2^ [86]. While clinical outcomes remained unchanged in both arms, serum p-cresol levels were significantly decreased in patients treated with the synbiotic. Cruz-Mora et al. followed 18 hemodialysis patients who received nutritional counseling before being divided into a test and control group [87]. Both gastrointestinal symptoms and fecal sample bacterial constitution were evaluated for 2 months; there was an increase in abundance of Bifidobacterium (*p* = 0.0344) and a reduction of symptoms in the treated group. A more recent RCT by Lopes et al. evaluated the effect of a synbiotic consisting of a mixture of unfermented milk and sorghum [88], showing that, compared to controls, CKD patients treated with the synbiotic had no significant changes in the URS measured.

In the SYNERGY study [89], synbiotic administration, consisting of a combination of resistant starch with nine taxa (six Lactobacilli, three Bifidobacteria, and Streptococcus thermophilus) did not affect the measured cytokines and did not reduce serum IS significantly, but there was a significant decrease in PCS concentrations. A sub-analysis of patients that were not treated with antibiotics during the study showed that synbiotic therapy resulted in a reduction in IS and PCS. However, a placebo-controlled RCT by McFarlane et al. (SYNERGY II) urged caution after the investigators witnessed a significant decrease of eGFR in patients receiving the synbiotic after 1 year [90]. As compared to the SYNERGY trial, a larger population of patients received a higher dose (20 mg daily) of a similar supplement for a longer period (1 year).

#### 4.3.4. Postbiotics

Finally, with the increased interest in postbiotics in the health community, the ISAPP defined them as “a preparation of inanimate microorganisms and/or their components that confers a health benefit on the host” [91]. By studying markers of lipid peroxidation in aging mice, the combination of probiotic and postbiotics had a high anti-oxidant effect in multiple organs including the kidneys [92]. As suggested by Cosola et al., their use in CKD will need to be evaluated in clinical studies [93].

**Table 1:** Supplements in clinical studies.

Tested Supplement	Study Authors	Design of Study	Number of Subjects	Intervention	Main Results of the Intervention
Prebiotics	Biruete et al. [94]	Crossover RCT	12 HD patients	Inulin	No statistically significant changes of measured URS
	Meijers et al. [71]	Non-randomized, open label phase I/II study	22 HD patients	Oligofructose-enriched inulin	Decreased PCS generation rates and serum concentration
	Raj et al. [72]	Non-randomized, crossover feasibility study	13 HD patients	Oligofructose-enriched inulin	➢ Modification of the gut microbiome ➢ No statistically significant changes of measured URS
	Li et al. [95]	Crossover RCT	21 PD patients	Inulin-type fructan	➢ No statistically significant changes of measured URS
	Poesen et al. [73]	RCT	40 CKD patients	Arabinoxylan Oligosaccharides	Decreased TMAO level but no impact on IS and PCS
	de Paiva et al. [74]	RCT	16 HD patients	Resistant starch	Reduced plasma level of biomarkers of inflammation
	Esgalhado et al. [96]	Crossover RCT	26 HD patients	Resistant starch enriched cookies	Decreased level of plasma IS
	de Andrade et al. [75]	Crossover RCT	43 PD patients	Unripe banana flour	No statistically significant changes of measured URS
	Ramos et al. [76]	RCT	50 CKD patients	Fructooligosaccharide	No statistically significant changes of measured URS
	Armani et al. [77]	RCT	46 CKD patients	Fructooligosaccharide	➢ Decreased interleukin-6 levels ➢ No statistically significant changes of measured URS
	Ebrahim et al. [97]	RCT	59 CKD patients	Beta-glucan	➢ Decreased levels of unbound IS, PCS, and PCG
Probiotics	Ranganathan et al. [82]	RCT	46 CKD patients	Probiotic bacterial formulation	➢ Decreased BUN ➢ Overall quality of life improvement
	Liu et al. [83]	RCT	50 HD patients	Probiotic bacterial formulation	➢ Significant IAA-O-glucuronide decrease ➢ No significant impact on microbiome diversity
	Lim et al. [84]	RCT	56 HD patients	Lactobacilli and Lactococcus	➢ Significant decrease of IS
Synbiotics	Guida et al. [86]	RCT	30 CKD patients	Probinul-neutro	➢ Decreased total plasma level of p-cresol
	Cruz-Mora et al. [87]	RCT	18 HD patients	Synbiotic	➢ Decrease in Lactobacillus ➢ Bifidobacterium species increase
	Lopes et al. [88]	RCT	99 CKD patients	Synbiotic	➢ No statistically significant changes of measured URS
	Rossi et al. [89]	RCT	37 CKD patients	High molecular weight inulin with conjugated oligosaccharides and 9 bacterial species	➢ Reduction in PCS level and decreasing plasmatic IS trend ➢ Bifidobacterium species increase
	McFarlane et al. [90]	RCT	68 CKD patients	Composition similar to the one used in Rossi et al. study	➢ Increased creatinine ➢ Bifidobacterium species increase
	Cosola et al. [98]	RCT	23 CKD patients and 27 healthy volunteers	Fructo-oligosaccharide, inulin, L casei, B animalis, and antioxidants	➢ Decreased unbound IS in CKD patients

BUN: blood urea nitrogen; CKD: chronic kidney disease; HD: hemodialysis; PD: peritoneal dialysis; RCT: randomized controlled trial.

### 4.4. Antibiotics Treatment

Antibiotics could provide an alternative to pre- and probiotic therapy to effectively alter the microbiome. In a study evaluating six patients receiving a combination of ciprofloxacin and metronidazole for 1 week [18], the investigators performed a phosphatidylcholine challenge before and after the administration of the antibiotic and then another challenge one month later. TMAO plasma concentrations almost disappeared after antibiotic treatment and reappeared after a month with great variability. In an RCT by Kimber et al. [99], 38 patients with eGFR < 60 mL/min/1.73 m^2^ were divided into two equal groups given a 10 day course of Rifamixin vs. placebo. The use of this oral non-absorbable antibiotic had no impact on the circulating TMAO, IS, or PCS. Beta-diversity of the gut microbiota assessed via fecal samples did not significantly change between study arms.

To date, the effect of oral vancomycin administration on URS plasma levels was evaluated in two studies. In a pilot study by Nazzal et al. [100], 10 hemodialysis patients received a single oral 250 mg vancomycin dose before stool and blood were regularly sampled. A significant decline in IS and PCS was observed on day four. IS and PCS levels returned to baseline after 28 days, while large population changes occurred at the genus level with a decrease in taxonomic richness. This study was followed by a randomized placebo-controlled trial in which vancomycin (250 mg) was administered once weekly for 12 weeks to subjects receiving thrice-weekly hemodialysis [101]. Subjects exhibited a decrease in 7 URS, including PCS and IS. Antibiotic administration significantly altered gut microbiota assessed using stool samples with a decrease in both alpha and beta diversity. A potential concern with the long term use of antibiotics is the development of bacterial resistance; however, prolonged antibiotics have been successfully used in other conditions including cystic fibrosis, bronchiectasis, and inflammatory bowel disease [102].

### 4.5. Enzymatic Modulation

Innovative therapeutic measures are targeting bacterial enzymatic pathways involved in the production of URS precursors. In a bioinformatic investigation, Popkov et al. investigated the bacterial enzymes contributing to URS formation in the human gut [103]. Using data from the Huma Microbiome Project 2 (HMP2), the authors analyzed sequences from the gut microbiome of 735 healthy individuals and found 173 of these enzymes. Nonlethal inhibition of bacterial enzymes is already being studied in several different enzymatic pathways.

An essential step in the pathogenesis of indolic compounds is the transformation of aromatic amino acids by bacterial tryptophanase [5]. An innovative therapeutic approach would be to target this enzyme in order to decrease IS plasma level by direct manipulation of the bacterial genome or post-translational modulation. After deletion of the genes coding for tryptophanase, Devlin et al. colonized germ-free mice with two types of Bacteroidetes (wild type or a mutant that does not produce indole from tryptophan) [104]. When comparing levels of IS in the urine, they found that mice colonized with the mutant bacteria had lower levels of IS after 5 and 9 days as compared to mice colonized with the wild-type bacteria. Post-translational protein modification was successfully achieved by Lobel et al. with the use of nutritional measures [105]; a diet enriched in amino acids with sulfur groups led to an increased bacterial tryptophanase S-sulfhydration, and this post-translational alteration inhibited indole production by Escherichia coli. Oikawa et al. explored the use of lignans extracted from sesame seeds as competitive inhibitors of tryptophanase in an in vitro study [106]. The kinetics measurement showed that (+)-sesamin, sesamol, and sesaminol efficiently inhibited this enzyme. Isoquercitrin, an ubiquitous sugar found in different foods and beverages, inhibited indole synthesis in vitro, possibly through decreasing bacterial uptake of tryptophan [107].T

A few studies evaluated the effect of inhibitors of the choline TMA-lyase on TMAO synthesis. 3,3-Dimethylbutanol (DMB) not only reduced enzymatic activity in intact and lysate of bacteria but also decreased TMAO levels in C57BL/6J and apolipoprotein E null mice (ApoE −/−) while both were on a choline supplemented diet [108]. DMB administration also lowered the percentage of foam cells in the studied mice and induced a beneficial shift in the taxa associated with TMAO. In a study by Witkowski et al., fluoromethylcholine (FMC) decreased TMAO thrombogenicity via a reduction in the plasma levels of TMA and TMAO [20]. Choline-fed mice receiving FMC had almost no aortic expression of tissue factor compared to control. Iodomethylcholine (IMC) reduced the level of TMAO and attenuated CKD progression in ApoE −/− mice fed adenine but failed to reduce atherosclerotic lesions in the aorta compared to mice fed adenine alone [109].

In the PS synthesis pathway, 2-aza tyrosine was used to disrupt bacterial TPL, and this compound had a beneficial effect on the kidneys, reducing both creatinine and albuminuria in mice without important alteration of alpha and beta diversity of their microbiome [14].

However, one should be cautious because some precursors of these URS have beneficial effects on the colon. For example, indole seems to play an important anti-inflammatory role in the gut. In an in vitro cell study of colonic cells, Bansal et al. were able to evaluate indole’s role in interkingdom interaction, witnessing a specific decrease in the activity of pro-inflammatory transcription factor NF-kB as well as decreased epithelial permeability [110]. A beneficial effect was also found in vivo with indole attenuating inflammation of the small intestine distal to the duodenum in mice receiving indomethacin for 1 week [111].

### 4.6. Other Therapeutic Options

Fecal microbiota transplantation (FMT) was attempted in an adenine-induced CKD mouse model by Barba et al. [112]. FMT significantly reduced phenol compounds but did not affect IS, IAA, and hippuric acid.

A hypothetical mechanism that could lead to an improved elimination of the protein-bound URS is through an increase of the free/bound ratio of these solutes. Kemp et al. explored fatty acids as potential competitors of IS and PCS to the binding sites of plasmatic carriers [113]; PCS was found to have a significant negative correlation with multiple polyunsaturated fatty acids.

Modulation of means of excretion could also become a new therapeutic option. Extensive research is being conducted to identify key solutes, receptors, and transporters involved in the “remote sensing and signaling” between the kidney and the gut and to understand how uremia disrupts this interorgan communication [114]. Jansen et al. described a communication system between microbiome derived URS, specifically IS receptors located in the renal proximal tubule and the OAT1 transporter [115].

Not only kidney but also gut transporters are being targeted in animal studies for reducing URS. While studying the effects of canagliflozin in CKD mice, Mishima et al. found that a two weeks treatment with this co-transporter inhibitor led to a significant reduction in plasma levels of PCS and IS with a modification of gut microbiota diversity [116]. An inhibitor targeted specifically at the gastro-intestinal SGLT1, named SGL5213, was able to reduce both PS and TMAO in an adenine-induced CKD mouse; attenuating kidney injury on histopathology [117]. This poorly absorbable compound also seemed to reduce dysbiosis, decreasing the Firmicutes/Bacteroidetes ratio.

## 5. Conclusions

The gut microbiome is essential in the generation of several uremic toxins. Although multiple therapeutic options are currently being explored, much progress is still required, whether it concerns the concomitant use of multiple options (for example, a combination of healthy diet, supplements, and antibiotic regimen) or the assessment of targeted modifications of microbiota to reduce the level or inhibit bacterial enzyme function in clinical studies.

With each patient’s microbiota being unique, personalized therapies might be needed to optimize the benefit from microbiome-targeted therapies.

Focusing on the long-term impact of the different therapies studied should be undertaken to assess a lasting impact on the metabolomic profile (i.e., a prolonged decrease of IS or PCS after a limited intervention). A longer follow-up could also be useful to screen for the emergence of bacterial resistance in studies evaluating antibiotics and their efficacy. Further, the evaluation of clinical outcomes is still scarce in literature; this step is necessary in order to link a decrease of URS and an eventual improvement of the cardiovascular risk profile through follow-up of endpoints such as CKD progression or cardiac morbidity/mortality. Finally, current and future potential therapies should not only assess the mere decrease of a uremic toxin level, but similarly to studies involving antibiotics and nonlethal inhibition of bacterial enzymes, they should evaluate the ecological consequences of their administration on the gut community and its diversity.

## Data Availability

Not applicable.
